# Development of a real-time reverse transcription loop-mediated isothermal amplification method for the rapid detection of porcine epidemic diarrhea virus

**DOI:** 10.1186/s12985-015-0297-1

**Published:** 2015-05-14

**Authors:** Xuewu Yu, Lin Shi, Xiaoping Lv, Wei Yao, Minghui Cao, Hanxun Yu, Xiurong Wang, Shimin Zheng

**Affiliations:** College of Veterinary Medicine, Northeast Agricultural University, No.59, Mucai street, Xiangfan District, Harbin, 150030 China; Animal Epidemic Diseases Control and Prevention Center of Liaoning Province, Shenyang, China; China Institute of Veterinary Drugs Control, Beijing, China; Animal Epidemic Diseases Control and Prevention Center of Dalian city, Dalian, China; Animal Influenza Laboratory of the Ministry of Agriculture and State Key Laboratory of Veterinary Biotechnology, Harbin Veterinary Research Institute, Chinese Academy of Agricultural Sciences, Harbin, China

**Keywords:** PEDV, RT-LAMP, Detection

## Abstract

**Background:**

Porcine epidemic diarrhea (PED) is an acute and highly contagious enteric disease characterized by severe enteritis, vomiting and watery diarrhea in swine. Recently, the outbreak of the epidemic disease has been a serious problem in swine industry. The objective of this study is to develop a rapid, sensitive, and real-time reverse transcription loop-mediated isothermal amplification (RT-LAMP) method for the detection of porcine epidemic diarrhea virus (PEDV) in less equipped laboratories.

**Results:**

The optimal reaction condition of the current real-time RT-LAMP for PEDV was 62 °C for 45 min. It was capable of detecting PEDV from clinical samples and differentiating PEDV from several related porcine viruses, while it did not require additional expensive equipment. The minimum detection limit of the real-time RT-LAMP assay was 0.07PFU per reaction for PEDV RNA, making this assay approximately 100-fold more sensitive than that of one-step RT-PCR. By screening a panel of clinical specimens, the results showed that this method presented a similar sensitivity with real-time RT-PCR and was somewhat sensitive than one-step RT-PCR in detection of clinical samples.

**Conclusions:**

In this study, we have developed a new real-time RT-LAMP method, which is rapid, sensitive and efficient to detect PEDV.This method holds great promises not only in laboratory detection and discrimination of PEDV but also in large scale field and clinical studies.

## Background

Porcine epidemic diarrhea (PED) is an acute, highly contagious and devastating enteric disease characterized by severe enteritis, vomiting and watery diarrhea in swine [1]. PED is caused by porcine epidemic diarrhea virus (PEDV), which was firstly identified in Belgium in 1978 [2]. PEDV is an enveloped, single-strand, and positive-sense RNA virus, which belongs to the Coronaviridae family [3]. The PEDV genome is ~28 kb in length and comprised of a 5′ untranslated region (UTR), a 3′ UTR, and at least seven open reading frames (ORFs) that encode four structural proteins [spike (S),envelope (E), membrane (M),and nucleocapsid (N)] and three non-structural proteins(replicase 1a and 1b, and ORF3) [4].

In China, PED was firstly occurred in Shanghai in 1973. So far, PEDV has been observed on most swine breeding farms in most provinces since late 2010 in China [5].The economic losses caused by PEDV infection have been continuous and serious in China [6]. Recently, PEDV has suddenly emerged in the United States and rapidly spread across the country, resulting in high mortality in infected newborn piglets, which have posed serious economic losses to the swine industry in the USA [7, 8]. Rapid diagnosis and timely monitoring of potential PED outbreaks are among the first important steps in the prevention and control of PED. Currently, several conventional methods are available for the detection of PEDV, including virus isolation, fluorescence assay, immune electron microscopy, enzyme-linked immuno- sorbent assay and molecular biological characterization [9]. However, the isolation and identification of viruses require extended periods of time ranging from days to weeks; so this method does not meet the time requirements needed for the prevention of epidemics. Therefore, these rapid, sensitive and specific molecular biological techniques, including RT-PCR and real-time RT-PCR, have played important roles in the rapid detection of PEDV [10]. Nevertheless, all of these techniques require sophisticated instrumentation (such as PCR machines and quantitative fluorescence PCR machines), limiting the effectiveness of these procedures in smaller, under-equipped laboratories.

The loop-mediated isothermal amplification (LAMP) technique is a molecular biology method used to amplify specific DNA fragments *in vitro* [11]. This method only requires a water bath or heating block to amplify large amounts of nucleic acids in 30 ~ 60 minutes without additional expensive equipments [12]. In addition, there is no need to use nucleic acid electrophoresis to assess the result, for the reason that the result can be easily observed in the presence of a fluorescent dye [13]. These characteristics make the LAMP method a simple, fast, effective and practical DNA amplification method, which has been successfully implemented for the detection of avian influenza A viruses [14], porcine reproductive and respiratory syndrome virus [15], foot-and mouth disease virus [16] and PEDV [17]. The PEDV M protein, the most abundant envelope component, is a triple-spanning membrane glycoprotein with a short amino-terminal domain outside of the virus and a long carboxy-terminal domain inside [18]. The M protein plays an important role in the virus-assembly process, and induces antibodies that neutralize the virus in the presence of its complement [19]. In this study, five primer sets were designed based on the conserved regions of the M gene, and a real-time RT-LAMP method was developed for the detection of PEDV.

## Results

### Primer set screening for real-time RT-LAMP assay

**Fig. 1:**
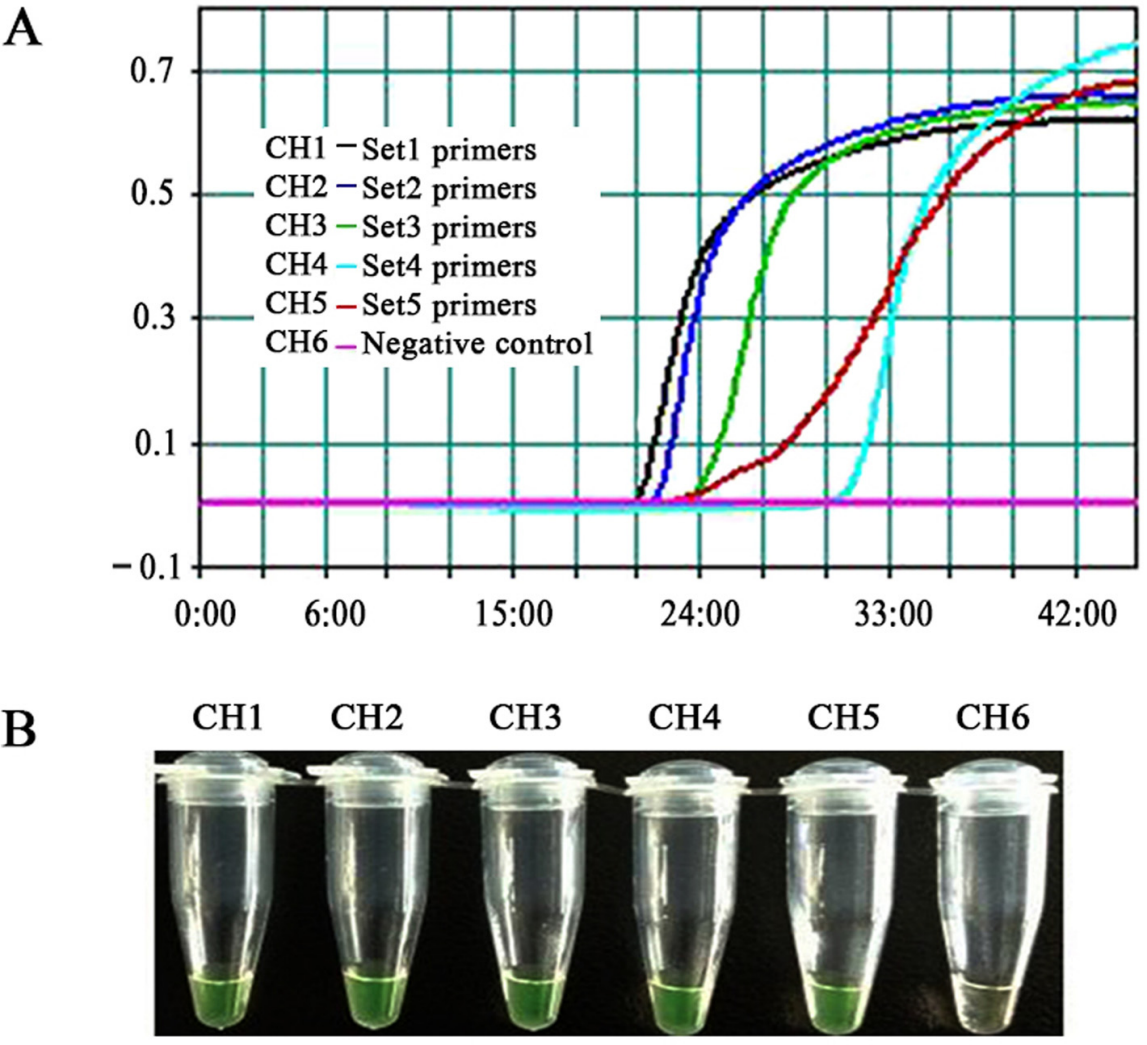
Primer set selection for the real-time RT-LAMP assay. LAMP products were detected by a real-time turbidity assay using an LA-320c (**a**) and a fluorescence assay (**b**)

To select the optimal primer set for the real-time RT-LAMP assay, the primer set screening assay was investigated at 63 °C for 45 min using the LA-320C Loopamp real-time turbidimeter (Teramecs, Japan). As show in Fig. 1a, the first primer set was the best one for the real-time RT-LAMP assay of PEDV among the five primer sets. However, in the end of the RT-LAMP amplification reactions, the real-time turbidity of the fifth primer set was somewhat higher than that of other primer sets; and there were no significant difference in the fluorescence intensities of the products among using these five primer sets (Fig. 1b). As a result, the first primer set was used in subsequent studies.

### Optimal temperature for real-time RT-LAMP assay

**Fig. 2:**
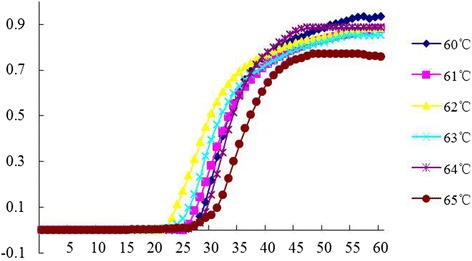
Optimization of temperature for the real-time RT-LAMP assay. The same reaction mixtures were incubated at 60, 61, 62, 63, 64, or 65 °C for 1 h, respectively

Using the first primer set, the effect of reaction temperature on the real-time RT-LAMP was investigated. As show in Fig. 2, the best temperature for the real-time RT-LAMP assay of PEDV was at 62 °C. However, in the end of the RT-LAMP amplification reactions, the real-time turbidity of DNAs from the reactions at 60 °C was somewhat higher than that at other reaction temperatures, but which was not determined as the optimal temperature for the real-time RT-LAMP amplifying PEDV M gene.

### Sensitivity of real-time RT-LAMP

**Fig. 3:**
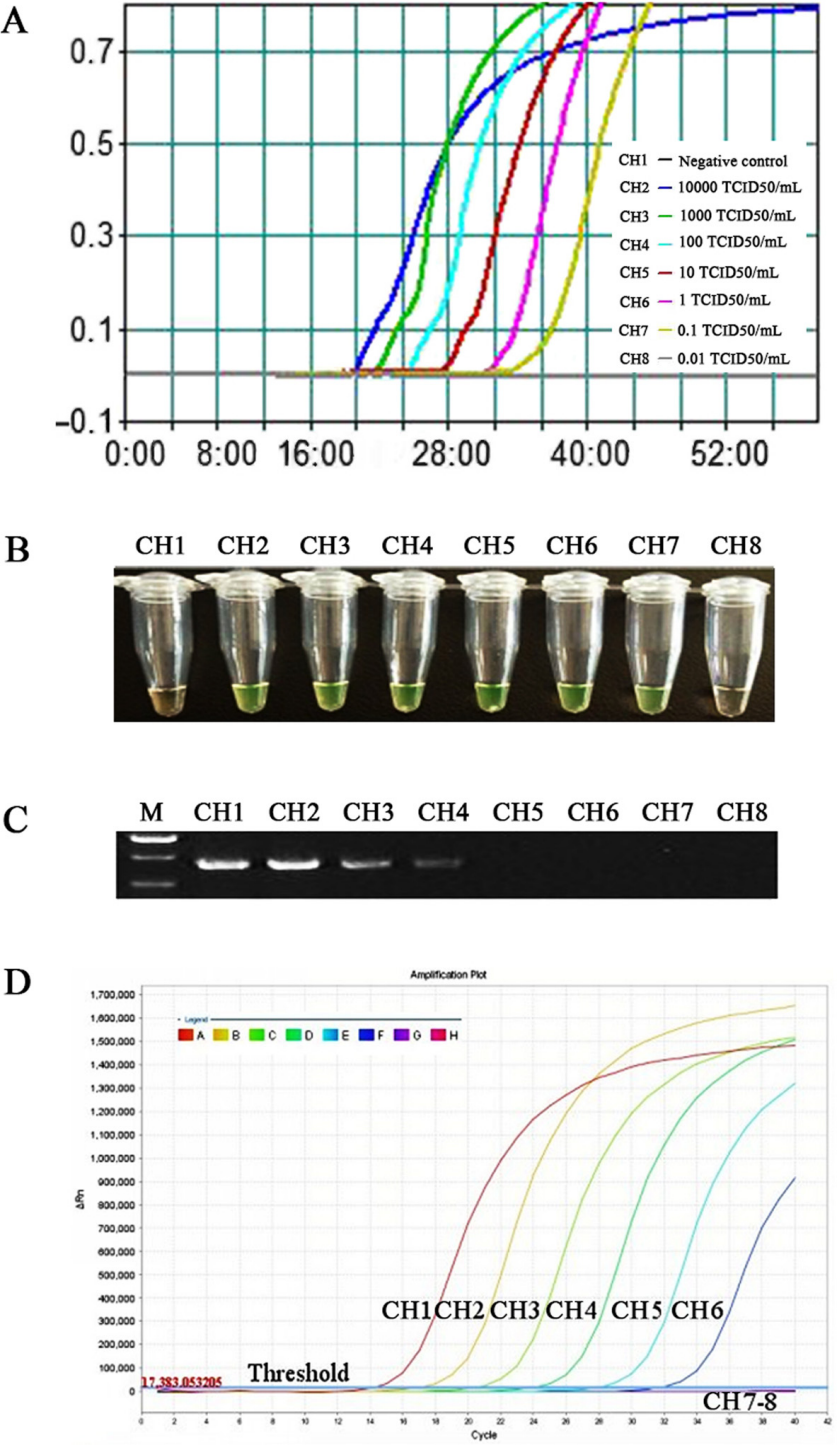
Comparative sensitivity of real-time RT-LAMP, one-step RT-PCR, and real-time RT-PCR methods. Real-time RT-LAMP, one-step RT-PCR and real-time RT-PCR were performed using viral RNAs at the concentrations ranging from 10^4.0^ to 10^−2^ TCID_50_/mL per tube. **a** and **b** Detection limit of real-time RT-LAMP. LAMP products were detected using a real-time turbidity assay with an LA-320c (**a**) and a fluorescence assay (**b**). **c** Detection limit for the one-step RT-PCR using the same RNA extracts that were used for real-time RT-LAMP. PCR products were observed in a 1.5 % agarose gel that was stained with ethidium bromide. The lanes from left to right were as follows: lane M, DNA marker DL2000; lanes 1 ~ 7, the one-step RT-PCR results from10^4.0^ to 10^−2^ TCID_50_/mL diluted virions; lanes 8, negative control. **d** Detection limit for real-time RT-PCR using the same RNA extracts that were used for real-time RT-LAMP. PCR products were detected using a real-time fluorescence assay with an ABI7500 system. Line 1–7, the real-time RT-PCR results from10^4.0^ to 10^−2^ TCID_50_/mL diluted virions; Line8, negative control

To evaluate the sensitivity of the real-time RT-LAMP assay, the detection limit of the assay was determined by testing 10-fold serial dilutions of the PEDV (LNsy201401), which has a defined median tissue culture infective dose (TCID_50_). The real-time RT-LAMP sensitivity assay was performed at 62 °C for 60 min. The kinetic analysis of the real-time turbidity revealed that the real-time RT-LAMP assay was able to detect the PEDV at the level of 10^−1^ TCID_50_/mL per tube, which equal to a virus titer of 0.07 PFU (Fig. 3a). The assay sensitivity was also confirmed by visual inspection. As shown in Fig. 3b, the clear fluorescence signals were observed at the concentrations ranging from 10^4.0^ to 10^−1^ TCID_50_/mL per tube. There were no differences observed in the sensitivity between the real-time turbidity and visual fluorescence detections that were associated with the real-time LAMP assay.

When the same RNA template was subjected to one-step RT-PCR and real-time RT-PCR, the detection limit of the one-step RT-PCR was 10^1.0^ TCID_50_/mL per tube, which equal to a virus titer of 7.0 PFU (Fig. 3c); while the detection limit of the real-time RT-PCR was 10^−1^ TCID_50_/mL per tube, which equal to a virus titer of 0.07 PFU (Fig. 3d). The results indicated that the sensitivity of the real-time RT-LAMP assay was approximately 100-fold higher than that of one-step RT-PCR, and the real-time RT-LAMP had a similar sensitivity as the real-time RT-PCR.

The results suggested that the real-time turbidity of DNAs from the reactions at concentrations ranging from 10^4.0^ to 10^−1^ TCID_50_/mL showed high intensities when the reaction was performed for 45 min (Fig. 3a). Therefore, the optimal reaction condition of the current real-time RT-LAMP for PEDV was 62 °C for 45 min.

### Specificity of real-time RT-LAMP

**Fig. 4:**
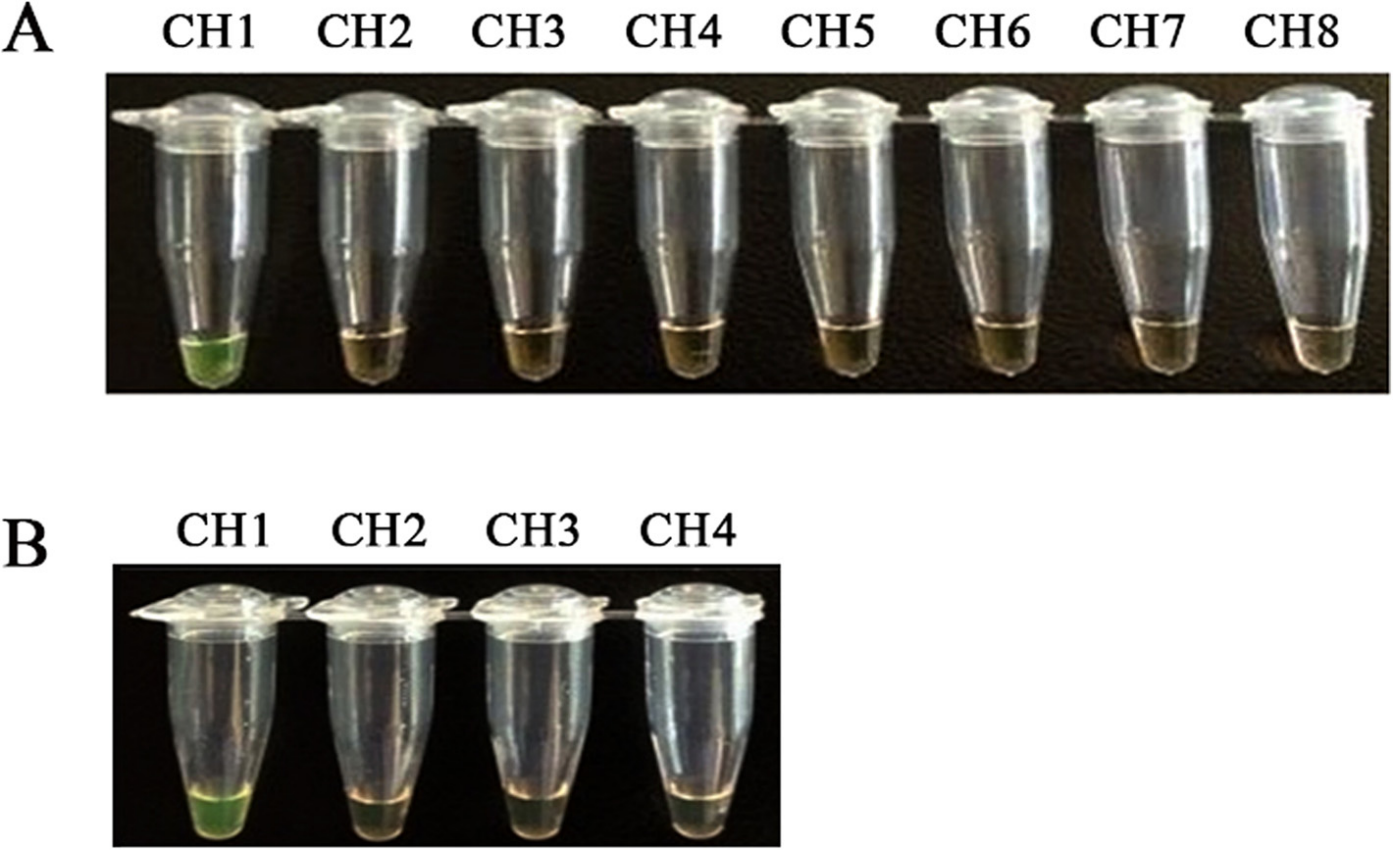
Specificity of real-time RT-LAMP assay for different porcine viruses. Viral RNA or DNA samples were extracted from different porcine viruses. The real-time RT-LAMP reaction was assessed based on visual inspection. **a**The channel 1–8 (CH1-CH8) were real-time RT-LAMP results from the templates PEDV, CSFV, PRRSV, TGEV, PRV, SIV(H1N1), PCV2, and negative control , respectively. **b** The channel from left to right were as follows: CH1, PEDV; CH2, PPV; CH3, PrV; CH4, negative control

Following the optimization of the conditions of the real-time RT-LAMP, several related porcine viruses (including CSFV, PRRSV, TGEV, PRV, SIV(H1N1), PCV2, PPV and PrV) were tested using the real-time RT-LAMP assay to evaluate the primer specificity. PEDV (LNsy201401) was used as the positive control, and the reactions that were performed in the absence of the template were used as negative controls. Only the PEDV was positive, and no LAMP products were detected in the reactions that were performed with RNAs or DNAs harvested from other relevant swine viruses used in this study (Fig. 4a and b). These results indicated that the real-time RT-LAMP assay was specific and can used to specifically amplify PEDV.

### Detection of PEDV in clinical samples

To evaluate the ability of the real-time RT-LAMP assay to detect PED viruses from clinical samples, 52 clinical samples were collected from ten pig farms in Liaoning province. All samples were tested by real-time RT-LAMP, one-step RT-PCR and real-time RT-PCR simultaneously. As shown in Table 1, real-time RT-LAMP gave 31 positive cases of 52 samples; one-step RT-PCR gave 27 positive cases and real-time RT-PCR gave 30 positive cases. The positive rates detected by real-time RT-LAMP, one-step RT-PCR and real-time RT-PCR were 59.6 % (31/52), 51.9 % (27/52) and 57.8 % (30/52), respectively. The real-time RT-LAMP had a similar sensitivity with real-time RT-PCR and was somewhat sensitive than one-step RT-PCR in detection of PEDV in clinical samples.

**Table 1:** Detection of PEDV in clinical samples by real-time RT-LAMP, real-time RT-PCR, and one-step RT-PCR

Types of samples	Total number of samples	PEDV positive samples		
		RT-PCR	real-time RT-PCR	RT-LAMP
Feces	37	18	20	21
Intestinal contents	15	9	10	10
Total	52	27	30	31

## Discussion

PEDV infection has caused continuous and severe economic loss in China. although inactivated vaccines against PEDV are used in some regions in China [5]. At present, the control of PEDV infection primarily depends on the early identification to prevent the further spread of the virus. Therefore, the development of a simple and rapid diagnostic method for PEDV is extremely significant. Even now, diagnostic methods for detecting PEDV all require high-precision instruments. Therefore, they are unsuitable for detecting PEDV in fields and in less well-equipped laboratories.

In this study, a real-time RT-LAMP assay with the PEDV M gene specific primers was successfully developed and optimized. The reaction conditions of the the real-time RT-LAMP were optimized by selecting primers sets and performing the test at different temperatures. Subsequently, its sensitivity was compared with that of one-step RT-PCR. The real-time RT-LAMP assay was able to detect PEDV with a detection limit of 10^−1^ TCID_50_/mL, which equals to a virus titer of 0.07 PFU (Fig. 3a). The 10^4.0^ TCID_50_/mL PEDV had an RNA viral load of 10^6.846^(7.02 × 10^6^) copies. The results showed that the specificity of real-time RT-LAMP for PEDV was approximately 100-fold more sensitive than that of one-step RT-PCR. Moreover, the sensitivity between real-time RT-LAMP and real-time RT-PCR was compared using the same PEDV RNA template. The real-time RT-LAMP had a similar sensitivity with real-time RT-PCR. Four clinical samples were determined to be PEDV negative by one-step RT-PCR, but the real-time RT-LAMP was able to detect them as PEDV positive. The results further suggested that the real-time RT-LAMP assay was slightly more sensitive than one-step RT-PCR for the detection of the PEDV M gene. The developed PEDV M gene real-time RT-LAMP method has a higher sensitivity (10^−1^ TCID_50_/mL) than the RT-LAMP method developed for N gene with detection limit 10^0.75^ TCID50/ml, which was determined to be more sensitive than gel-based RT-PCR and ELISA in previous study [17].

The real-time RT-LAMP method developed using this primer set was used to examine PEDV and eight additional porcine pathogens. The viruses such as CSFV, PRRSV, TGEV, PRV, SIV(H1N1), PCV2, PPV or PrV may cause co-infection in pigs; PEDV and TGEV belong to the Group I coronaviruses which are closely related [20]. The results showed that this method specifically amplified only the PEDV M gene sequences without any other porcine virus genes demonstrating that the real-time RT-LAMP possesses a high level of specificity for PEDV.

In conclusion, the real-time RT-LAMP method for PEDV established in this study demonstrated a high level of sensitivity and specificity. With the real-time RT-LAMP method, the results can be read visually in the absence of a real-time turbidimeter. The isothermal conditions required for LAMP can be provided with a conventional water bath or heat block, which can be applied in less well-equipped laboratories and fields for rapid detection of PEDV. Thus, compared to the requirements associated with one-step RT-PCR and real-time RT-PCR, the simplicity and affordability of the LAMP assay allow for the most convenient identification of PEDV among the three methods. This method offers a simple, effective, rapid and economical early diagnostic technique for the detection and potential control of PEDV.

## Conclusions

We have developed an efficient approach to rapidly detect PEDV. This method holds great promises not only in laboratory detection and discrimination of PEDV but also in large scale field and clinical studies.

## Materials and methods

### Viruses

PEDV LNsy201401 was isolated from intestinal tissue of piglets; and its M gene was sequenced (TaKaRa, Dalian, China). PEDV was propagated in African green monkey kidney (Vero) cells according to reference with modification [21, 22]. The median tissue culture infective dose per milliliter (TCID_50_/ml) of PEDV was 10^5.0^. Porcine reproductive and respiratory syndrome virus (PRRSV), classical swine fever virus (CSFV), transmissible gastroenteritis virus (TGEV), porcine rotavirus (PRV), porcine circovirus type 2 (PCV2), porcine parvovirus (PPV), and pseudorabies virus (PrV) were propagated in susceptible cells. Swine influenza virus (SIV, H1N1) was propagated in the allantoic sac and amniotic cavity of 9-day-old specific pathogen-free (SPF) chicken embryos for 48 to 72 hours at 37 °C [23, 24].

### Primers

Based on the M gene sequence of PEDV (JX435310), five sets of primers were designed using the Primer Explorer version 4 software (Eiken Chemical Co., Ltd., Tokyo, Japan; http://primerexplorer.jp/elamp4.0.0/index.html) and synthesized by Shanghai Sangon Co., Ltd. A set of primers included two outer primers (forward primer M-F3 and reverse primer M-B3), two inner primers (forward inner primer M-FIP and reverse inner primer M-BIP). For comparative purposes, conventional RT-PCR methods based on N and M genes were both constructed. The developed PEDV N gene conventional RT-PCR method sensitivity was higher, in comoparison to the conventional RT-PCR developed for M gene. Therefore, the pair of primers based on N gene (JQ743655, named P1 and P2) designed to amplify a 428-bp fragment was utilized in further study. The primers and probes used for real-time RT-PCR targeting N gene were listed in Table 2 [25]. The specificity of the primers for real-time RT-LAMP was confirmed against random nucleotide sequences using a BLAST search in GenBank databases located in the National Center for Biotechnology Information (NCBI, http://www.ncbi.nlm.nih.gov/BLAST/).

**Table 2:** Sequences of the primers and probe used in this study

Application	Primer name	Length(bp)	Primer/probe sequence(5′ to 3′)
Set 1 primers for RT-LAMP	M1-F3	20	GGACACATTCTTGGTGGTCT
	M1-B3	20	CCAACACGTCCGTAGACAAT
	M1-FIP	42	TGGTGCTCCAAGCACTGGAATGACGCGCTTCTCACTACTTCT
	M1-BIP	40	AAGGTTGCTACTGGCGTACAGGTTGTAGTGGCCTTGGCGA
Set 2 primers for RT-LAMP	M2-F3	18	ACAGACGCGCTTCTCACT
	M2-B3	20	CCAACACGTCCGTAGACAAT
	M2-FIP	39	AGCGTTACACCAGTTGGTGCTCTTCTGTGATGGGCCGAC
	M2-BIP	40	AAGGTTGCTACTGGCGTACAGGTTGTAGTGGCCTTGGCGA
Set 3 primers for RT-LAMP	M3-F3	19	GCGCAGGACACATTCTTGG
	M3-B3	19	TTGGCGACTGTGACGAAAT
	M3-FIP	40	GGAATGCAGACCTGTCGGCCTCAATCCTGAAACAGACGCG
	M3-BIP	41	TGGAGCACCAACTGGTGTAACGGTACGCCAGTAGCAACCTT
Set 4 primers for RT-LAMP	M4-F3	18	CCGACAGGTCTGCATTCC
	M4-B3	20	CCAGTGCCAGATGAAGCATT
	M4-FIP	42	CCTGTACGCCAGTAGCAACCTTGAGCACCAACTGGTGTAACG
	M4-BIP	41	ATTTCGTCACAGTCGCCAAGGCGACTGAACGACCAACACGT
Set 5 primers for RT-LAMP	M5-F3	20	AGCTTTCAGGTCAATTGGGT
	M5-B3	20	GGAGTGTTAGCGTTACACCA
	M5-FIP	42	TGCGCCACAACCGAATGCTATTCAGCATCCTTATGGCTTGCA
	M5-BIP	41	TCAATCCTGAAACAGACGCGCTTGCTCCAAGCACTGGAATG
RT-PCR	P1	21	TTCCCAGCGTAGTTGAGATTG
	P2	21	CGAAGTGGCTCTGGATTTGTT
real-time RT-PCR	PED-NF	24	CGCAAAGACTGAACCCACTAATTT
	PED-NR	24	TTGCCTCTGTTGTTACTTGGAGAT
	PED-Cy5	24	Cy5-TGTTGCCATTGCCACGACTCCTGC-BHQ3

### RNA extraction

The total RNAs were extracted from the culture supernatants of PEDV, CSFV, PRRSV, TGEV, PRV, SIV(H1N1) using the TRIzol reagent (Invitrogen, CA, USA) and the genomic DNAs of PCV2, PPV and PrV were extracted with the DNAzol reagent (Invitrogen, CA, USA) according to the manufacturer’s instructions. The extracted RNA or DNA was resuspended in 20 μL of diethylpyrocarbonate water or sterile water.

### Real-time RT-LAMP

The real-time RT-LAMP reaction was performed in a final reaction volume of 25 μL by using a Loopamp RNA amplification kit (Eiken Chemical Co. Ltd., Japan) containing 1 μL RNA template, 40 pmol each of inner primers M-FIP and M-BIP, 5 pmol each of outer primers M-F3 and M-B3, 1.4 mM dNTPs mix, 0.8 M betaine, 0.1 % Tween20, 10 mM (NH_4_)_2_SO_4_, 8 mM MgSO_4_, 10 mM KCl, 20 mM Tris–HCl (pH 8.8), 16 U Bst DNA polymerase (New England Biolabs, USA), 0.125 U AMV (Invitrogen, CarlsBad, CA, USA) and 1 μL fluorescent detection reagent (FD) (Eiken Chemical Co. Ltd., Japan).

Amplification reactions were performed at 63 °C for 60 min using either a LA-320C Loopamp real-time turbidimeter (Teramecs, Japan) or in a water bath. The mixtures were heated at 80 °C for 10 min to terminate the reactions. The turbidity of the reaction was measured in real time, and the result was indicated by the graph on the monitor of real-time turbidimeter, verifying the start of the amplification. LAMP products were then evaluated with a fluorescent detection reagent (Eiken Chemical Co., Ltd., Japan). A negative control (a sample devoid of template) was included in each reaction.

### Optimization of the real-time RT-LAMP assay

To determine the optimal reaction temperature, the real-time RT-LAMP reaction mixtures were incubated at 60, 61, 62, 63, 64, or 65 °C for 1 h. The optimal reaction time was determined by performing the RT-LAMP real-time sensitivity assay at the optimal temperature.

### RT-PCR and real-time RT-PCR

RT-PCR was performed with primers (P1 and P2) specific for the PEDV N gene using a PrimeScript™ one-step RT-PCR kit(Takara, Dalian, China). RT-PCR conditions were optimized. RT-PCR parameters included 50 °C for 30 min, 94 °C for 2 min, 35 cycles at 94 °C for 30 s, 55 °C for 30 s, 72 °C for 40 s, followed by the final extension at 72 °C for 2 min. The RT-PCR products were subjected to electrophoresis on a 1.5 % agarose gel, and the target bands were visualized by staining with ethidium bromide.

The real-time RT-PCR was performed with the primers (PED-NF and PED-NR) and the probe (PED-Cy5) specific for the N gene of PEDV, as described previously [20]. The quantitative one-step RT-PCR kit (Invitrogen Life Technologies™, USA) was used for real-time RT-PCR. In brief, real-time RT-PCR was carried out in a 20 μL reaction containing 0.8 μL of ThermoScript™ plus/ Platinum® Taq Enzyme Mix, 10 μL of 2× ThermoScript Reaction Mix (a final concentration of 3 mM MgCl_2_), 0.5 μL of both PEDV forward and reverse primer, 0.5 μL of PEDV-Cy5 probe, 2 μL of RNA, and 5.7 μL of water. The reaction was carried out in an ABI7500 (Applied Bio systems) under the following conditions: initial reverse transcription at 58 °C for 30 min, followed by initial denaturation at 95 °C for 5 min, 40 cycles of denaturation at 95 °C for 30 s, and annealing and extension at 60 °C for 1 min. The intensities of the fluorescent dyes in each reaction were read automatically during PCR cycling and optical data were analyzed with 7500 software v2.0.6.

### Detection of PEDV in clinical samples by one-step RT-PCR, real-time RT-PCR, and real-time RT-LAMP

In total, fifty-two clinical samples (including feces and intestinal samples) from piglets with signs of severe watery diarrhea, dehydration were collected from ten pig farms in Liaoning province in China between February 2014 and June 2014. The samples were homogenized with phosphate-buffered saline (PBS, pH 7.4) as a 10 % (w/v) suspension and centrifuged for 10 min at 1700× g at 4 °C. The supernatant were collected and stored at −80 °C until used. The supernatant was subjected to RNA extraction with above-mentioned RNA extraction kit. The resulting RNA was used as a template for one-step RT-PCR, real-time RT-PCR and real-time RT-LAMP according to above-mentioned protocols.
